# On the principle of reciprocity in inelastic electron scattering

**DOI:** 10.1107/S2053273324009550

**Published:** 2024-10-21

**Authors:** Budhika G. Mendis

**Affiliations:** a Department of Physics Durham University South Road Durham DH1 3LE United Kingdom; Helmholtz Centre for Infection Research, Germany

**Keywords:** principle of reciprocity, inelastic scattering, entropy

## Abstract

In electron microscopy the principle of reciprocity is often used to imply time reversal symmetry. While this is true for elastic scattering, its applicability to inelastic scattering is less well established. From the second law of thermodynamics, the entropy for a thermally isolated system must be constant for any reversible process. Using entropy and statistical fluctuation arguments, it is shown that, while reversibility is possible at the microscopic level, it becomes statistically less likely for higher energy transfers. The implications for reciprocal imaging modes, including energy loss and energy gain measurements, as well as Kainuma’s reciprocal wave model are also discussed.

## Abstract 2

This article examines the principle of reciprocity in inelastic scattering using entropy-based criteria.

## 1. Introduction

The principle of reciprocity states that source and detector positions can be interchanged without altering the measured intensity. Originally formulated for light optics, it is also widely used in electron microscopy, for example to explain the similarities between conventional and scanning transmission electron microscopy imaging (Findlay *et al.*, 2003 ▸; Krause & Rosenauer, 2017 ▸), crystal structure analysis using convergent-beam electron diffraction (Buxton *et al.*, 1976 ▸) and coherence volume in high-angle annular dark-field imaging (Treacy & Gibson, 1993 ▸). These studies combined reciprocity with time reversal symmetry, which is only strictly valid for elastic scattering (Kohl & Rose, 1985 ▸). In fact, time reversal symmetry is a sufficient but not necessary condition for reciprocity (Bilhorn *et al.*, 1964 ▸; Sigwarth & Miniatura, 2022 ▸).

**Figure 1:**
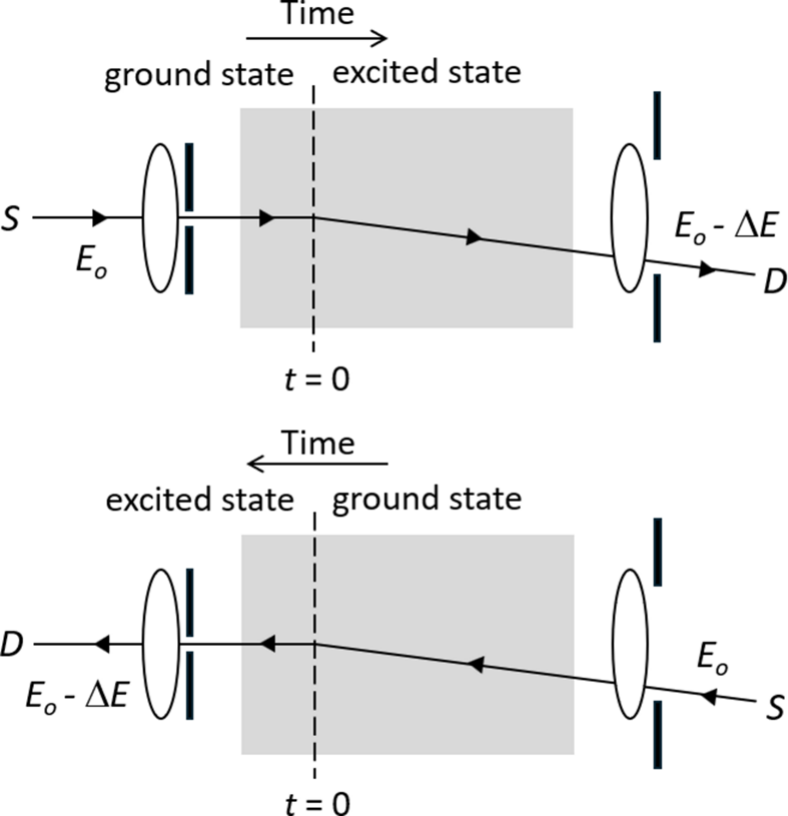
Two reciprocal imaging modes, where the position of the source (S) and detector (D) are swapped. The incident electron with primary energy *E_o_* undergoes an inelastic scattering event at time *t* = 0 (energy loss Δ*E*) within the specimen (grey box). The specimen is in the ground state prior to inelastic scattering.

**Figure 2:**
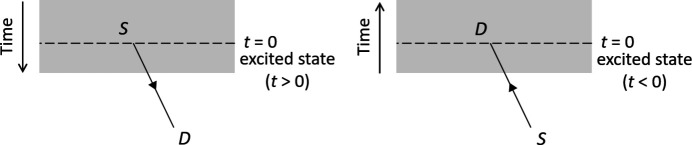
Time reversal symmetry in Kainuma’s reciprocal wave model. Left: the source (S) is an inelastic scattering event at time *t* = 0 occurring within the solid (grey box). Following inelastic scattering the specimen is in an excited state. Right: the reciprocal wave model swaps source and detector (D) positions, while the electron trajectory is also reversed.

As an example, Fig. 1 ▸ shows schematic reciprocal imaging modes for inelastic scattering measurements. Time reversibility can only be approximately satisfied if the energy loss 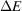 is sufficiently small to have negligible effect on the lens aberrations and transmission of the high-energy electron within the sample and vacuum (Pogany & Turner, 1968 ▸; Kohl & Rose, 1985 ▸; Findlay *et al.*, 2007 ▸). Time reversal symmetry is also implicitly assumed in the reciprocal wave model of Kainuma (1955 ▸), which is widely used to simulate the angular distribution of inelastic scattering (Fig. 2 ▸), including thermal diffuse scattering (Kainuma, 1955 ▸; Alanazi *et al.*, 2023 ▸), core loss excitations (Rusz *et al.*, 2017 ▸) and Compton scattering (Mendis & Talmantaite, 2022 ▸). This short communication explores time reversibility and reciprocity in the context of inelastic scattering, using classical thermodynamics arguments. It is shown that the probability of satisfying reversibility decreases exponentially with energy transfer. Implications for reciprocal imaging modes and reciprocal wave simulations are also discussed.

## 2. Thermodynamic analysis

Consider a thermally isolated system consisting of a single, high-energy electron propagating through a much larger specimen. The latter is modelled as a thermal reservoir at temperature 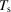. A temperature 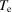 can also be assigned to the electron, based on the statistical definition of temperature, *i.e.*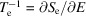, where 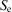 and *E* are the entropy and energy of the electron, respectively (Blundell & Blundell, 2010 ▸). Since 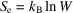, it is required to count the number of available microstates *W* (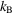 is Boltzmann’s constant). An electron with wavenumber *q* can have a wavevector terminating anywhere on its Ewald sphere. If the measurement accuracy of the wavenumber is 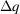, then 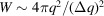. Several factors may contribute to 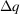, such as the position-momentum uncertainty principle and energy spread of the electron emitter. As we shall see, the value of 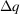 is not important for the present discussion. The electron energy is given by 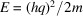, where *h* is Planck’s constant and *m* is the electron mass. It follows that 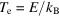. The temperature of the high-energy electron (typically 200 keV in transmission electron microscopy) is much larger than that of the specimen, which is assumed to be at room temperature. The ‘hot’ electron can therefore exchange some of its energy with the specimen, signifying inelastic scattering.

Consider an inelastic event with energy transfer 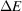 from the electron to the specimen. From the first law of thermodynamics, the change in internal energy of the electron (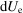) and specimen (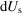) is given by
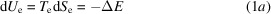

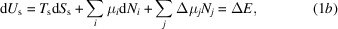
where 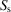 is the entropy for the specimen. The summation over *i* describes the creation of 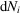 number of quasi-particles during inelastic scattering, each with chemical potential 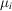. Examples include phonons (Forbes *et al.*, 2010 ▸), plasmons (Mendis, 2019 ▸) and magnons (Mendis, 2022 ▸). The second summation describes promotion of 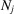 number of solid-state electrons to higher energy states (*e.g.* core loss excitations), accompanied by a change in chemical potential 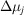. According to the second law of thermodynamics, the total entropy change 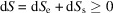, the equality applying for reversible processes. We find
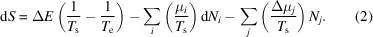
Since 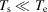, equation (2) simplifies to
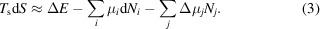


Since the number of phonons, plasmons or magnons is not conserved, 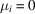 (Blundell & Blundell, 2010 ▸), so that the *i* summation can be ignored. The chemical potential is by definition the free energy per particle, and hence the *j* summation is equal to the change in specimen free energy 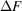 (both Gibbs and Helmholtz free energies are equal in this case, due to constant volume and pressure). Therefore
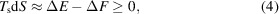
where we have used the fact that 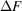, which is the maximum available energy for doing work (Blundell & Blundell, 2010 ▸), cannot be greater than 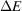 due to energy conservation.

It may appear that inelastic scattering is always irreversible because thermal energy cannot be transferred from the cooler specimen to the higher-temperature incident electron. While this is certainly true for macroscopic systems, it is not a strict requirement at the microscopic level, such as individual inelastic scattering events. In fact, equation (4) does not necessarily rule out reversibility and, furthermore, phonon energy gain has been experimentally measured using electron energy loss spectroscopy (Lagos & Batson, 2018 ▸). Thermal fluctuations allow the specimen to be momentarily in an excited state, and consequently transfer its excess energy 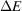 to the incident electron. From the Boltzmann distribution, the probability that the specimen is in an excited state is proportional to 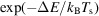. It follows that fluctuations, and therefore reversibility, are less likely for larger energy transfers. This can also be explained quantum-mechanically using the energy–time uncertainty principle. Larger values of 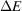 have shorter collision times, so that the quasi-static conditions required for reversibility are less likely to be achieved. Dissipation can occur via perturbations to the neighbouring electrons and nuclei in the solid, such as, for example, during screening of the core hole (Mendis & Ramasse, 2021 ▸) following core loss excitation.

## 3. Discussion

Implications for reciprocal imaging modes and Kainuma’s (1955 ▸) reciprocal wave model will now be discussed. For the former, the typical experimental setup illustrated in Fig. 1 ▸ does not satisfy time reversibility, although mathematically this does not preclude reciprocity. Many authors (Pogany & Turner, 1968 ▸; Kohl & Rose, 1985 ▸; Findlay *et al.*, 2007 ▸) have suggested that reciprocity and time reversibility are approximately satisfied, provided the energy loss is small. On the other hand, energy loss and energy gain images will be identical, since the entire system (*i.e.* sample and optical elements) is time reversible, a sufficient condition for reciprocity (Sigwarth & Miniatura, 2022 ▸). Note that the lower signal observed in practice for energy gain compared with energy loss (Lagos & Batson, 2018 ▸) is not a violation of reciprocity. It is simply a result of the sample being less likely to be in the excited state compared with the ground state.

Next consider Kainuma’s (1955 ▸) reciprocal wave. As shown in Fig. 2 ▸, the source is now an inelastic scattering event, so that the specimen must be in an excited state for strict time reversibility of the reciprocal wave. However, in practice, this criterion is unnecessarily restrictive, and it is reasonable to assume the sample is in the ground state for the reciprocal wave. For example, core loss events are highly localized to a single atom and its immediate environment, and do not perturb the rest of the (bulk) solid. Plasmons, on the other hand, are more delocalized, but do not significantly alter the overall scattering potential, which consists of all electrons and atomic nuclei (Mendis, 2019 ▸). The sample may also rapidly decay back to the ground state, especially for high energy losses, which further supports ignoring excited-state effects. Therefore, the reciprocal wave effectively satisfies time reversibility, provided it only undergoes elastic scattering along its path. This does not however preclude any inelastic scattering of the reciprocal wave; rather these events will be excluded by energy filtering, leaving only the elastic reciprocal wave. If the energy resolution of the filter is limited, then time reversibility is only approximately satisfied.
